# Temporal and sequential order of nonoverlapping gene networks unraveled in mated female Drosophila

**DOI:** 10.26508/lsa.202101119

**Published:** 2021-11-29

**Authors:** Claude Pasquier, Alain Robichon

**Affiliations:** 1 Université Côte d’Azur, CNRS, I3S, Nice, France; 2 Université Côte d’Azur, INRAE, CNRS, ISA, Nice, France

## Abstract

In this study, we reanalyzed available datasets of gene expression changes in female Drosophila head induced by mating. Mated females present metabolic phenotypic changes and display behavioral characteristics that are not observed in virgin females, such as repulsion to male sexual aggressiveness, fidelity to food spots selected for oviposition, and restriction to the colonization of new niches. We characterize gene networks that play a role in female brain plasticity after mating using AMINE, a novel algorithm to find dysregulated modules of interacting genes. The uncovered networks of altered genes revealed a strong specificity for each successive period of life span after mating in the female head, with little conservation between them. This finding highlights a temporal order of recruitment of waves of interconnected genes which are apparently transiently modified: the first wave disappears before the emergence of the second wave in a reversible manner and ends with few consolidated gene expression changes at day 20. This analysis might document an extended field of a programmatic control of female phenotypic traits by male seminal fluid.

## Abstract (precis)

Mating triggers successive waves of temporal transcriptomic changes within independent gene networks in female Drosophila, suggesting a recruitment of interconnected modules that vanish in late life.

## Introduction

Phenotypic plasticity is associated with changes in gene expression and can be adaptive to fluctuating environmental conditions or nonadaptive independent of the context of natural selection pressure. Many acquired phenotypic traits appear irreversible in individuals of many insect species (DeWitt et al, 1998; Debat & David, 2001; Braendle & Felix, 2008, 2009; Dingemanse et al, 2010; Beldade et al, 2011). However, in insects like *Drosophila*, phenotypic plasticity operating in female after mating can produce the apparition of behavioral and physiological traits that can be reversible in relation to tissue specific temporal changes of transcriptome (Fedorka et al, 2007; Dalton et al, 2010; Ameku & Niwa, 2016; Delbare et al, 2017). Briefly, the female post-mating response includes increased egg laying and feeding (Carvalho et al, 2006), a preference for amino acids and for salt (Walker et al, 2015), decreased receptivity to mating (Chapman et al, 2003), decreased daytime sleep (Isaac et al, 2010; Garbe et al, 2016; Dove et al, 2017), and a diminished immune response (Short & Lazzaro, 2010, 2013). Moreover, in *Drosophila* species, mated females show an increase in activity during the light phase compared with virgin females and the same low level activity in the dark (Isaac et al, 2010). An increased consumption of yeast in the post-mating period was observed that corresponds to intense oogenesis (Drummond-Barbosa & Spradling, 2001). Another report describes that some genes of the immune system was highly expressed after mating, in contrast with a severely diminished efficiency of the fly to fight infectious pathogens (Fedorka et al, 2007). These conclusions have been completed by another observation that presents a panel of modified genes in mated female, most of them involved in immune system (Lawniczak & Begun, 2004). All these elements argue in favor of complex gene networks under the control of seminal fluid that markedly change the female destiny.

At physiological level, mating was reported to modify the gustatory signaling leading to an increase in appetite and uptake of salt and yeast by activation of Sex Peptide Receptor located in neuronal terminals in female reproductive tract (Walker et al, 2015). In this regard, few male seminal peptides that regulate general female metabolism, behavior, and life span have been identified to act in post mating period (Peng et al, 2005; Isaac et al, 2010). The strength of female gametogenesis is regulated by environmental factors, temperature, food abundance, and also mating. Stem cell division in ovary was found partly under control of male-derived Sex Peptide (SP) acting through a neuroendocrine pathway, neuromediators, and signaling molecules (Ameku & Niwa, 2016).

Authors have equally reported time-dependent changes at transcriptional level in mated female (Mack et al, 2006; Dalton et al, 2010). In a related study, ion channel transcripts have been found significantly down-regulated, whereas most of up-regulated genes reside in the head fat body (Dalton et al, 2010). Furthermore, mating that changes the physiology and behavior of *Drosophila* female was shown to affect the secretory granule release at some pre synaptic nerve terminal spaces in reproductive female tract (Heifetz & Wolfner, 2004). On the other side, an article reports that not only some mRNAs but microRNAs and histone chemical modifications are markers associated with mating leading to the idea that an epigenetic component orchestrate the genes networks recruitment (Zhou et al, 2014). Moreover, strong differences in female post-mating transcriptome has been reported depending on female/male genetic interactions, which suggests a solid contribution of genetic background to modulate the post mating female physiology (Delbare et al, 2017). The gene expression changes in female head and central nervous system tissues that follow mating and seminal acquisition has been documented in some extent. Behavior changes induced as post mating response was shown to be dependent on neuronal circuitry requiring expressing of doublesex (dsx) gene (Rezával et al, 2012). Authors have shown that the changes in gene expression induced by mating are influenced by cross tissue interactions and the global effects impact sleep, food preference, and re-mating (Newell et al, 2020). Robust changes in mRNA patterns in tissues between virgin and mated female have indicated integrative and coordinated functional gene networks acting in a concerted manner upon mating (McDonough-Goldstein et al, 2021). However, our knowledge on the integrative and coordinated process involving numerous transcriptional changes triggered by mating in female *Drosophila* have not been fully completed. At present, we know little about the molecular mechanisms that affect female brain gene expression after mating.

In this study, we use a new approach to analyze the transcriptomic changes occurring in the female head after mating. This approach relies on (1) a novel method, AMINE (Pasquier et al, 2021 *Preprint*), for the identification of dysregulated gene modules, and (2) accurate RNA-Seq data from the head of both virgin and mated *Drosophila* females at three time periods after mating, made available by the modENCODE project. AMINE applies a greedy algorithm on a highly informative and compact representation combining general knowledge about gene interactions and measured gene variation. This strategy allows us to assess both the extent of gene variation and the topology of gene interactions, overcoming limitations of classical enrichment analysis of the most differentially expressed genes and earlier network-based approaches.

Our current analysis, along with many other reports, argues in favor of a new paradigm that underlies a coordinated process of female transcriptome modification after mating, which parallels the behavioral and metabolic changes to produce offspring. The broad transcriptional changes initiated by copulation can be followed by the differential landscape of gene modules that vary between the three post-mating periods (1, 4, and 20 d after mating). Assuming that the long distance between the head and spermathecae excludes sperm RNA contamination during the experimental process of dissection, the analysis uncovered waves of modified gene networks that show marked differences between the time periods of 1, 4, and 20 d. This hints that mating triggers temporal and ordered activation of gene networks, that is, the first wave inducing the interdependent second wave and so on up to the end of the process.

## Results

### Differential expression analysis

**Figure 1.**
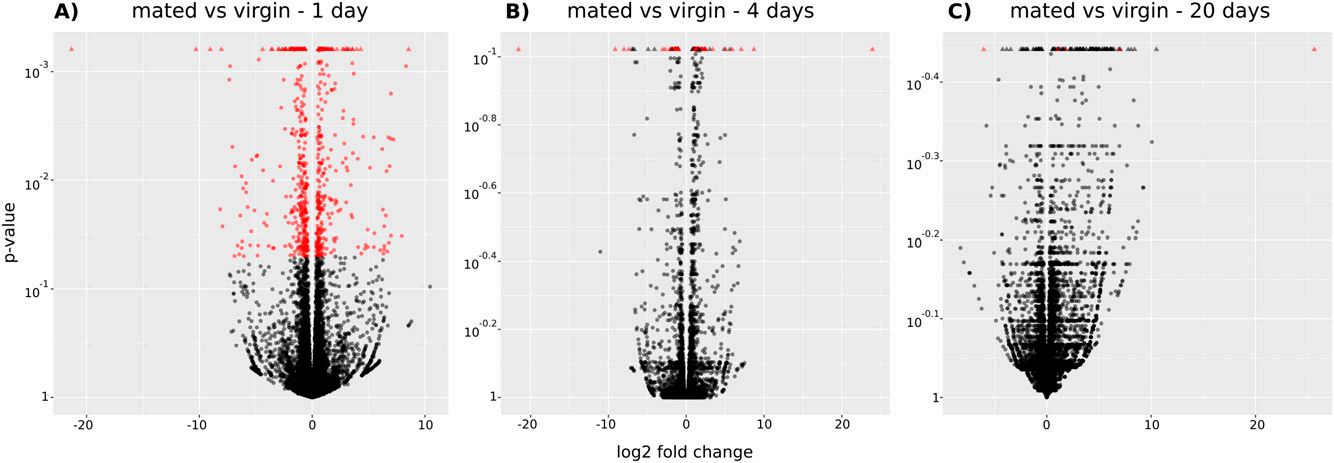
Volcano plots of differentially expressed genes. Volcano plots showing the significance of the differential expression of genes between mated and virgin flies at day 1, 4, and 20. Log_2_ FC is plotted on the x-axis, and adjusted *P*-values are plotted on the y-axis. Genes with a variation of at least a factor of 1.25 (i.e., a log_2_-fold change greater than 0.32 or less than −0.32) and an adjusted *P*-value of 0.05 or less are considered differentially expressed and are represented in red. Triangles correspond to genes with a too-high adjusted *P*-value to be displayed on the plot or a *P*-value equal to NA. Genes appearing in the three plots are listed in Tables S1–S3. **(A)** Mated versus virgin females at day 1. The analysis highlighted 625 differentially expressed genes identified with high confidence (most of these genes are associated with an adjusted *P*-value well below 0.05). **(B)** Mated versus virgin females at day 4. Eighty-two genes are considered differentially expressed. Important variations were found between replicates because most of the adjusted *P*-values were higher than 0.1, as seen in the panel. **(C)** Mated versus virgin females at day 20. At this time period, the large variations between the quantifications performed with the different replicates only allowed the identification of six differentially expressed genes.

**Figure 2.**
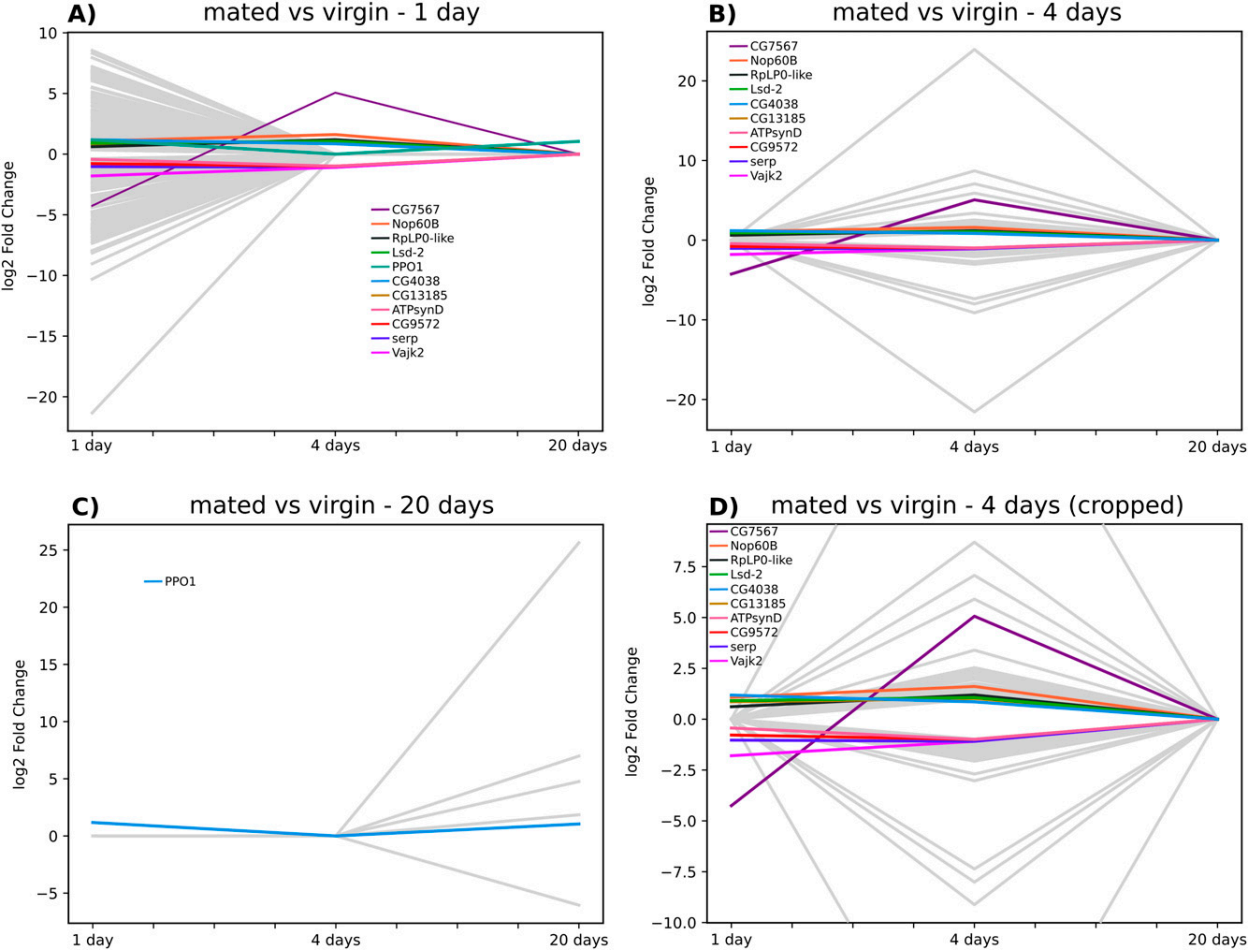
Line graph showing the evolution of log_2_ FC values over the three time points (days 1, 4, and 20) for genes that were identified as differentially expressed. **(A)** Evolution of log_2_ FC values for all genes identified as differentially expressed at day 1. The 11 colored lines correspond to the genes that were also differentially expressed at day 4 or 20. **(B)** Evolution of log_2_ FC values for genes identified as differentially expressed at day 4. The colored lines represent the genes that were also differentially expressed at any of the other time points. **(C)** Evolution of log_2_ FC values for genes identified as differentially expressed at day 20. The blue line illustrates the log_2_ FC value of PPO1, which was the only gene that was also overexpressed at day 1. **(C, D)** Cropped version of the panel shown in (C), which allows better differentiation among the different colored lines.

We considered only the RNA-Seq lists of head tissues of virgin versus mated females aged 1, 4, and 20 d, which eliminates any possible fertilized egg interference/contamination that occurs with the full carcass or other tissues, such as the gut. Under these conditions, the number of differentially expressed genes, which was at a maximal value 1 d post-mating (334 down-regulated and 291 up-regulated genes–Fig 1A and Table S1), decreased after 4 d (32 down-regulated and 50 up-regulated genes–Fig 1B and Table S2) until a very low number was observed after 20 d (one down-regulated and five up-regulated genes–Fig 1C and Table S3). The lists obtained for the three different time points mainly do not overlap. A single gene (FBgn0283437, prophenoloxidase 1 involved in the melanization reaction, notably in response to wounding and produced by crystal cells, a type of hemocyte in *Drosophila*) that is overexpressed at day 1 is also overexpressed at day 20 (Fig 2C). This gene is also known to enhance the mating process and reproductive success (Parkash et al, 2011). Only 10 genes were differentially expressed on both days 1 and 4 in the same orientation (Fig 2A–D). Of those, three genes were overexpressed in mated females (FBgn0030608/Lsd-2, FBgn0259937/Nop60B, and FBgn0032538/Vajk2), six were underexpressed (FBgn0260653/serp, FBgn0031089/aspr, FBgn0016120/ATPsynD, FBgn0033485/RpLP0-like, FBgn0033661/CG13185, and FBgn0011824/CG4038) in mated females, and one gene was overexpressed at day 1 but underexpressed at day 4 (FBgn0039670/CG7567). Little is known about the functions of this later gene, but the other nine genes are mainly implicated in rRNA processing and ribosome biogenesis. Fig 2 shows the evolution over the three time points of the log_2_-fold change (log_2_FC) values of the genes that were identified as differentially expressed at least at one time point.

Table S1 List of differentially expressed genes between virgin and mated females at day 1.

Table S2 List of differentially expressed genes between virgin and mated females at day 4.

Table S3 List of differentially expressed genes between virgin and mated females at day 20.

### Enrichment analysis of differentially expressed genes

Enrichment analysis is a classical process to obtain a synthetic view of a gene list. It consists in associating to this list the most typical annotations originating from various databases. We therefore performed enrichment analyses for the differentially expressed genes identified at each time point. We did this for all differentially expressed genes, for underexpressed genes, and then for overexpressed genes (results are listed in Table S4).

Table S4 List of enrichments of the genes differentially expressed at days 1, 4, and 20.

Annotations characterizing the whole list of differentially expressed genes at day 1 are associated with relatively modest false discovery rates (FDRs) on the order of 10 × 10^−4^. Among these annotations, we find several terms related to development (in particular cuticle development with a FDR of 1 × 10^−4^ and chitin-based cuticle development with a FDR of 1.5 × 10^−4^) and the chemical reactions involving carbohydrates (carbohydrate metabolic process with a FDR of 8.7 × 10^−4^). By considering only overexpressed genes, we find annotations associated with much more significant FDR. Among them, many annotations are related to metabolic regulation and cell cycle. Among the annotations associated with underexpressed genes, we find again terms related to cuticle development and carbohydrate metabolism. Regarding day 4, we find new annotations that did not appear significantly in day 1 as rRNA processing (FDR = 2.3 × 10^−7^) and ribosome biogenesis (FDR = 3.19 × 10^−7^). We find these same annotations, with even more significant FDR, for the underexpressed genes, whrereas no annotation appears significantly for the overexpressed genes. Concerning the six genes that are differentially expressed at day 20, no term from Gene Ontology stands out significantly. We only obtain an overrepresentation of the term Oxidoreductase from UniProt Keywords.

This transcriptome analysis highlights the observation that mating modifies the female biology and that these modifications affect temporal and sequential gene networks such that these show variations among days 1, 4, and 20. The number of genes with changes in the transcription level decreased markedly throughout the life span of female *Drosophila* after mating. Moreover, the analysis of the transcriptome at the three time periods revealed the specificity of the modified genes for each period, as revealed by the little overlap or conservation of these changes throughout the female fly life span after mating. This finding argues in favor of singular waves of gene networks acting at each time period after mating with little, if any, interference and acting as a cascade in which the first wave orchestrates the outcome of the second and so on along the fly life span.

### Limitations of conventional RNA-Seq data analysis pipeline

Differential gene expression analysis allows identification of the genes that vary the most. In clinical and pharmaceutical research, these genes can be valuable in identifying candidate biomarkers, therapeutic targets, and genetic signatures for diagnosis. However, the identification of specific changes in the expression of a few genes is usually not sufficient to reveal ongoing biological activities. The classical approach consisting in performing an enrichment analysis of the most differentially expressed genes does indeed hardly provide an accurate picture of the processes at stake. This is mainly due to the fact that in this approach, genes are considered as isolated entities. However, interactions between genes, is fundamentally important to understand the genetic pathways involved and the dynamics of complex genetic systems. As pinpointed by Rapaport et al (2007), “a small but coherent difference in the expression of all the genes in a pathway should be more significant than a larger difference occurring in unrelated genes.” Arising from this observation, many methods have been proposed for the analysis of gene activity based on our knowledge about their molecular interactions (Nguyen et al, 2019). The idea underlying active module identification is to identify pertinent modules of genes by simultaneously considering two criteria: one is based on a measurement of gene activity (differential expression) and the other reflects their molecular interactions. In their 2019 survey, Nguyen et al (2019) identified 22 computational tools for active module identification that they divided into six categories: greedy algorithms (six methods), evolutionary algorithms (five methods), diffusion-flow emulation models (five methods), random walk algorithms (two methods), maximum clique identification (two methods), and clustering-based methods (two methods). To this list, we add WMAXC (Amgalan & Lee, 2014), a method belonging to the category of maximum clique identification, GiGA (Breitling et al, 2004), which is based on a greedy algorithm and MRF (Robinson et al, 2017), which uses a diffusion-flow emulation model. This makes 25 methods, whose basic principles and results differ, but which also have differences from the point of view of usability; some are easy to use by a non-expert, others are more demanding, involving command line execution, complex setup, the need to properly format input data in a particular layout or requiring many steps to obtain the final result. Usability is a very important aspect for programs intended to be used by people who are not necessarily experts in computer science or data analysis. Difficulties may arise during installation, data preparation, or program execution.

#### Installation

A local installation requires downloading a package and installing it; it may require installing other required libraries or entering some command lines. Applications in the form of a web service allow access to a program simply by accessing a web address on a browser. A special case is represented by applications that come in the form of a plugin for Cytoscape (Shannon et al, 2003), a popular platform dedicated to network visualization and analysis. Cytoscape plugins require the use of Cytoscape but have the advantage of being easily installed through Cytoscape app store.

#### Data preparation

A module identification program requires two essential data: an interaction network and the quantification of the differential expression of genes which can be specified either by the level of expression in each condition, by a fold change (or a log_2_ fold change) or by a *P*-value. The interaction network is provided either by the application or by the user. If it is included in the application, it must be possible to link the identifiers of the genes or gene products whose expression has been measured to the identifiers of the nodes of the network. In the case where the interaction network is to be provided by the user, it usually takes the form of a list of edges, that is, a list in which each line contains a connection between the identifier of a source node and the identifier of a target node, possibly associated with a numerical value that may represent the strength of the interaction or its plausibility. All other requested parameters are detrimental to usability, whether they are internal program parameters or information about the desired results (e.g., number of modules and minimum and maximum module size).

#### Program execution

The most pleasant way to execute a program is to be in front of a graphical interface that allows us to enter the various input data. However, an execution in the form of commands to be entered on a command line interface, although less user-friendly, seems quite accessible. We consider programs that require numerous pre-processing steps, for example, for data conversion or parameter evaluation, to be useable only by computer-literate people and therefore not suitable for an end user.

Tables 1 and S5 show a summary of the methods emphasizing their availability and ease of use. Our study reveals that, out of the 25 methods, three are no longer accessible (broken link), one has problems in the installation procedure (installation failure), one causes a runtime error, two seem to run but do not return any results, two of the methods identified by Nguyen et al (2019) are not suitable for processing transcriptomic data and one does not allow the analysis of Drosophila data. There is also the case of three methods, which are available for free but must run in the MATLAB environment which is a commercial product. This makes a total of 13 non-useable methods which are identified by rows with white background in Tables 1 and S5. In these tables, eight methods that are potentially useable but not in an easy way for a non-specialist are displayed with an orange background. The difficulties come, for half of the cases, from the running of the applications in a software environment that has become obsolete: two methods work with java five which was released in 2004 (Java is currently in version 11) with a graphical interface based on Swing whose development was abandoned in 2014; two methods are plugins for Cytoscape two whose latest version dates from 2012. It might be possible to run these applications by reinstalling old versions of Java or Cytoscape, but one can expect to face difficulties in running these programs on recent software environments. In any case, these applications cannot be considered easy to use, so they have not been tested in our review. The four other programs considered as being difficult to use require the execution of several commands or transformations on the data which require a fair amount of work. At the end of this filtering, there are only four methods that an end user can use: JActiveModules (Ideker et al, 2002), the oldest method that comes as a plugin for Cytoscape 3, GiGA (Breitling et al, 2004) an application written in Perl, ClustEx2 (Gu et al, 2010), an application written in C++ and DIAMOnD (Ghiassian et al, 2015), an application written in Python 2. These last three applications must be run from the command line, but their invocation is straightforward. It should be noted that DIAMOnD identifies only one set of genes involved, unlike other methods that identify several modules. JActiveModules requires specification of an additional parameter which is the number of modules to identify. The three other methods require indicating the maximum size of the modules to be identified.

**Table 1.** Computational tools for active module identification.

name	Year	Reference	Type	Note
JActiveModules	2002	Ideker et al (2002)	Cytoscape plugin	OK
GiGA	2004	Breitling et al (2004)	cmd	OK
SAMBA	2004	Tanay et al (2004)	gui/cmd	Execution error
GXNA	2007	Nacu et al (2007)	cmd	Broken link
MATISSE	2007	Ulitsky and Shamir (2007)	gui	Difficult to use^a^
PinnacleZ	2007	Chuang et al (2007)	Cytoscape plugin	Difficult to use^b^
CEZANNE	2009	Ulitsky and Shamir (2009)	gui	Difficult to use^a^
BioNet	2010	Beisser et al (2010)	cmd	Difficult to use^c^
RegMOD	2010	Qiu et al (2010)	cmd	Not free to use^d^
ClustEx2	2010	Gu et al (2010)	cmd	OK
RME	2011	Miller et al (2011)	cmd	Not for transcriptomics data^e^
COSINE	2011	Ma et al (2011)	cmd	Difficult to use^f^^,^^g^
EnrichNet	2012	Glaab et al (2012)	cmd	Unable to run on drosophila data
MEMo	2012	Ciriello et al (2012)	cmd	Difficult to use^h^
BMRF-net	2013	Chen et al (2013)	Cytoscape plugin	Difficult to use^b^
Walktrap-gm	2013	Petrochilos et al (2013)	cmd	Installation failure
TimeXNet	2014	Patil and Nakai (2014)	web/gui/cmd	No results^i^
WMAXC	2014	Amgalan and Lee (2014)	cmd	Broken link
DIAMOnD	2015	Ghiassian et al (2015)	cmd	OK^g^
Hotnet2	2015	Leiserson et al (2015)	cmd	Difficult to use^j^
GLADIATOR	2017	Silberberg et al (2017)	cmd	Not for transcriptomics data^k^
MOEA	2017	Chen et al (2017)	cmd	Not free to use^d^
MRF	2017	Robinson et al (2017)	cmd	Not free to use^d^
ModuleDiscoverer	2018	Vlaic et al (2018)	cmd	Broken link
ResponseNet	2019	Basha et al (2019)	web	No results^i^
AMINE	2021	Pasquier et al (2021) *Preprint*	web	OK

a Program written in Java 5 with a graphical interface based on Swing whose development was abandoned when Java 8 appeared in 2014.

b Not available for Cytoscape 3.

c Require to execute a dozen of lines to process the example data included with the program.

d Need MATLAB which is not free.

e The method identifies patterns of recurrent genomic aberration in tumor samples.

f Requires to execute multiple commands in R language, in particular it is necessary to compute a matrix containing the differential correlation between each pair of connected nodes on the network.

g Identifies only one module.

h The documentation contains the following warning: Generating the files needed by the program may require a fair amount of work, as several of them are the results of other algorithms.

i The program ran for several hours without providing any results.

j Requires to execute multiples commands ; the script used to analyze data of the associated paper is 68 lines long.

k The aim of the program is to identify relationship between diseases modules ; user need to suply known genes associated with diseases.

Table S5 Detailed list of computational tools for active module identification reviewed in the article.

The commonality between all the existing methods is that their efficiency is highly dependent on the network topology. For these methods, two genes or gene products are as likely to be part of the same functional module as they are close on the interaction network. This proximity is described by the existence of an interaction or a chain of interactions between the two molecules. Unfortunately, molecular interaction networks stored in public databases are known to be noisy and incomplete (Wang et al, 2009). It has also been shown that PPI networks are “small-world” networks, meaning that the neighbors of a given node are likely to be neighbors of each other, and most nodes can be reached from every other node by a small number of hops.

Over the past few years, network embedding has emerged as a powerful network analysis approach by generating a highly informative and compact vector representation for each vertex in the network (Cui et al, 2018). This approach maps nodes into a vector space in which the distances between nodes accurately reflect their proximity in the original network. We hypothesize that network embedding methods can provide the basis for a more reliable method by estimating distances between nodes that take into account the entire topology of the graph and that in addition are little affected by the proportion of missing edges. Based on this assumption, we have developed AMINE, a new efficient method for detecting active modules in the vector space generated by an embedding of the interaction network (Pasquier et al, 2021 *Preprint*).

### Identification of active modules

**Figure 3.**
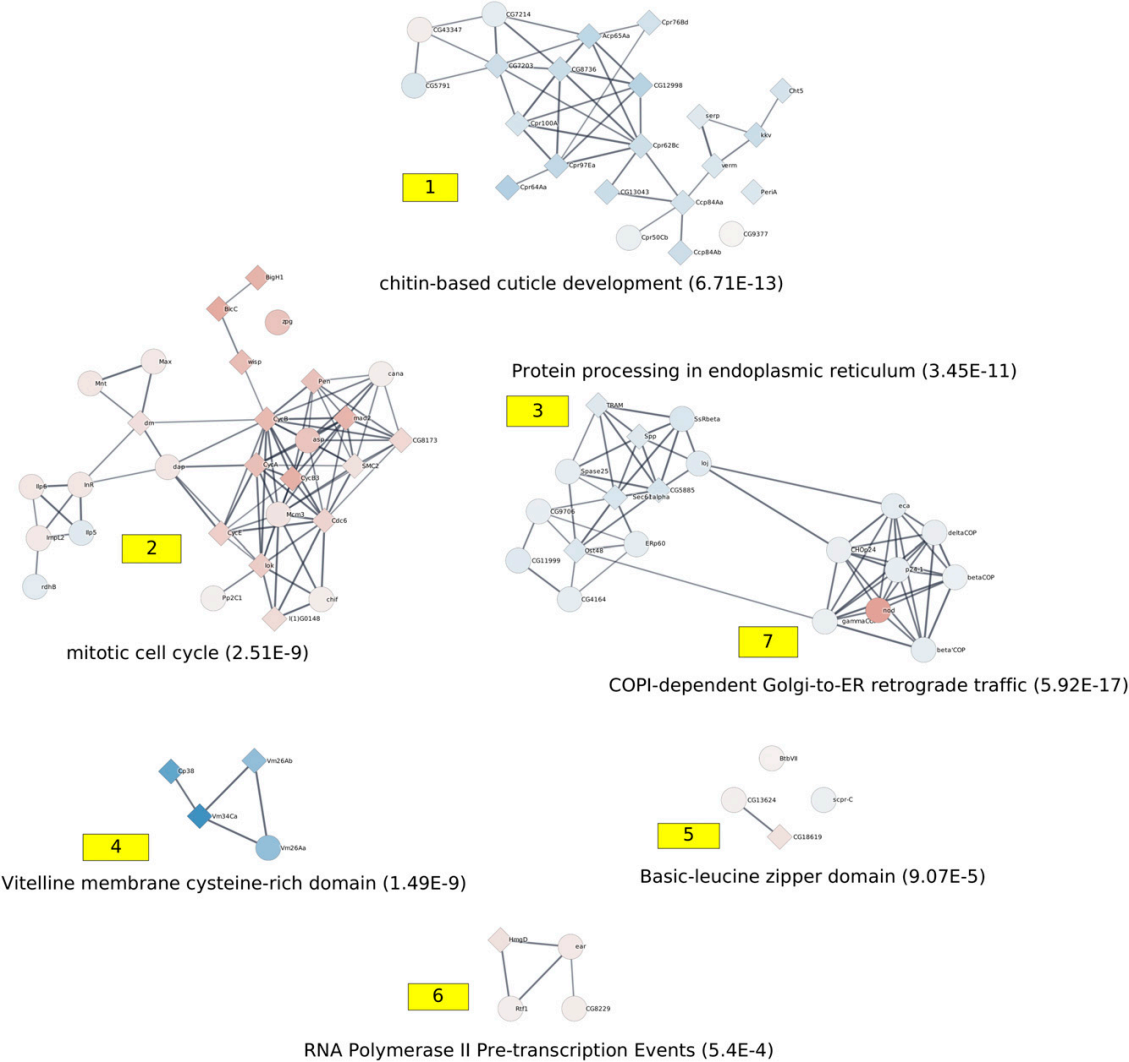
Representation of active modules identified at day 1. On the networks, the nodes correspond to genes, and the edges correspond to interactions reported in the String database with an evidence score ≥0.7. The number associated with each module corresponds to the module ID specified in Table S6. Each module is annotated with a representative enrichment. The complete lists of the enrichments of all the modules are shown in Table S9. The node colors represent the log_2_ FC values of the corresponding gene on a scale varying from blue (for the most underexpressed genes) to red (for the most overexpressed genes). The diamond-shaped nodes represent genes that are considered differentially expressed based on the DESeq2 method.

**Figure 4.**
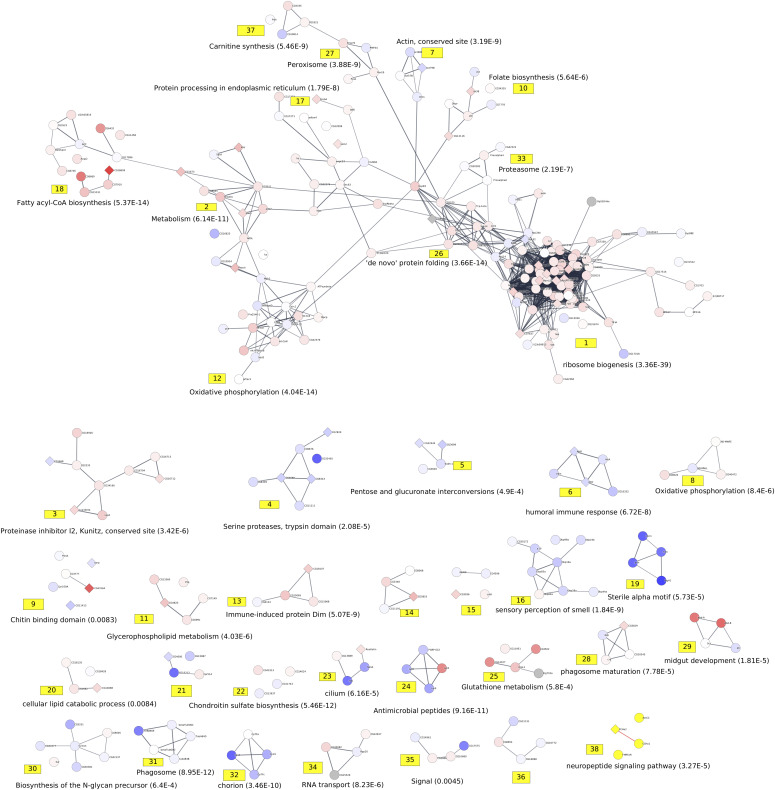
Representation of active modules identified at day 4. On the networks, the nodes correspond to genes, and the edges correspond to interactions reported in the String database with an evidence score ≥0.7. The number associated with each module corresponds to the module ID specified in Table S7. Each module is annotated with a representative enrichment (when available). The complete lists of the enrichments of all the modules are shown in Table S10. The node colors represent the log_2_ FC values of the corresponding gene on a scale varying from blue (for the most underexpressed genes) to red (for the most overexpressed genes). The diamond-shaped nodes represent genes that are considered differentially expressed based on the DESeq2 method.

**Figure 5.**
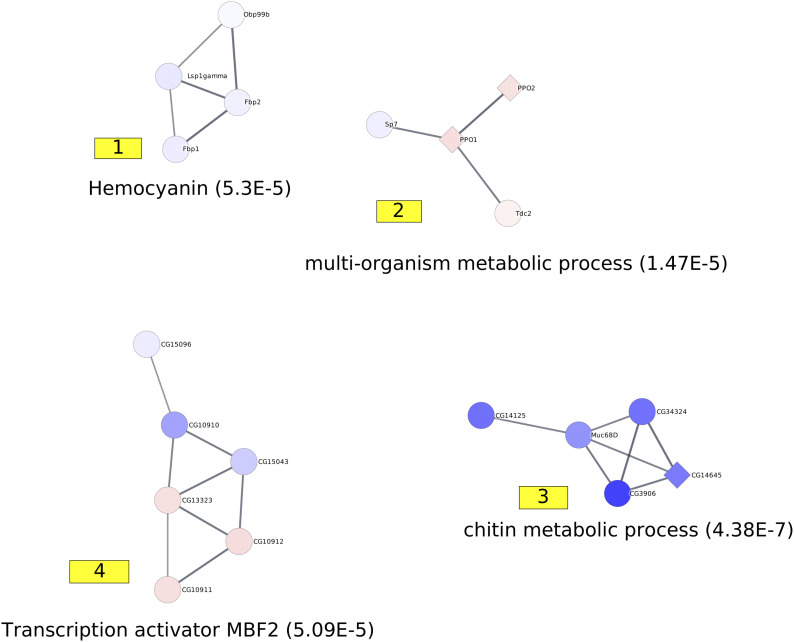
Representation of active modules identified at day 20. On the networks, the nodes correspond to genes, and the edges correspond to interactions reported in the String database with an evidence score ≥0.7. The number associated with each module corresponds to the module ID specified in Table S8. Each module is annotated with a representative enrichment. The complete lists of the enrichments of all the modules are shown in Table S11. The node colors represent the log_2_ FC values of the corresponding gene on a scale varying from blue (for the most underexpressed genes) to red (for the most overexpressed genes). The diamond-shaped nodes represent genes that are considered differentially expressed based on the DESeq2 method.

Using the web server at http://amine.i3s.unice.fr to execute the method AMINE, we proceeded to identify active modules in an independent manner for each of the three time points. Our method identified seven active modules of genes at day 1, 38 active modules at day 4 and four active modules at day 20 (Figs 3–5 and Tables S6–S8).

Table S6 List of genes belonging to the active modules identified at day 1.

Table S7 List of genes belonging to the active modules identified at day 4.

Table S8 List of genes belonging to the active modules identified at day 20.

**Figure 6.**
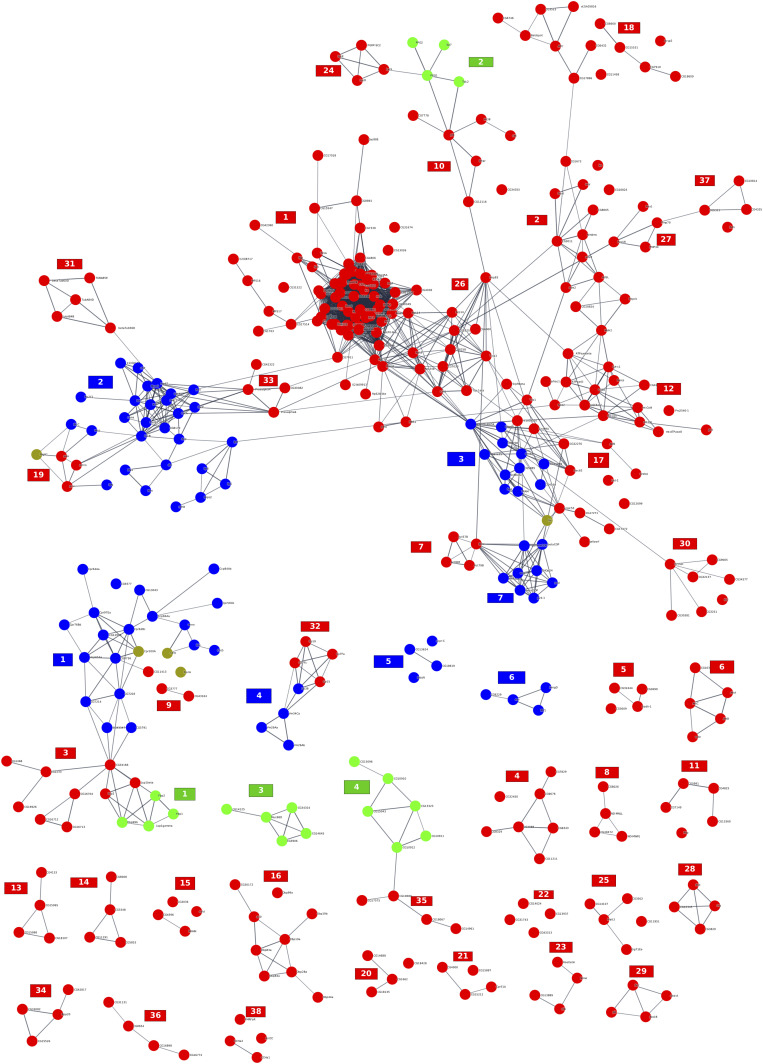
Union of the active modules identified at each time point. The network combines the active modules identified at days 1 (in blue), 4 (in red), and 20 (in green). The module numbers correspond to those specified in Figs 3–5.

Drawing the modules identified at the different time points on the same picture showed few overlapping genes (only five genes, FBgn0038252/BigH1, FBgn0260653/serp, FBgn0061492/loj, FBgn0039805/Cpr100A, and FBgn0022770/PeriA, which belong to modules identified at days 1 and 4) but highlights the fact that many modules interact between the different time points. Fig 6 depicts a network that expands from seven to 38 modules from day 1–4 and then shrinks to the four modules that are still active at 20 d.

**Figure 7.**
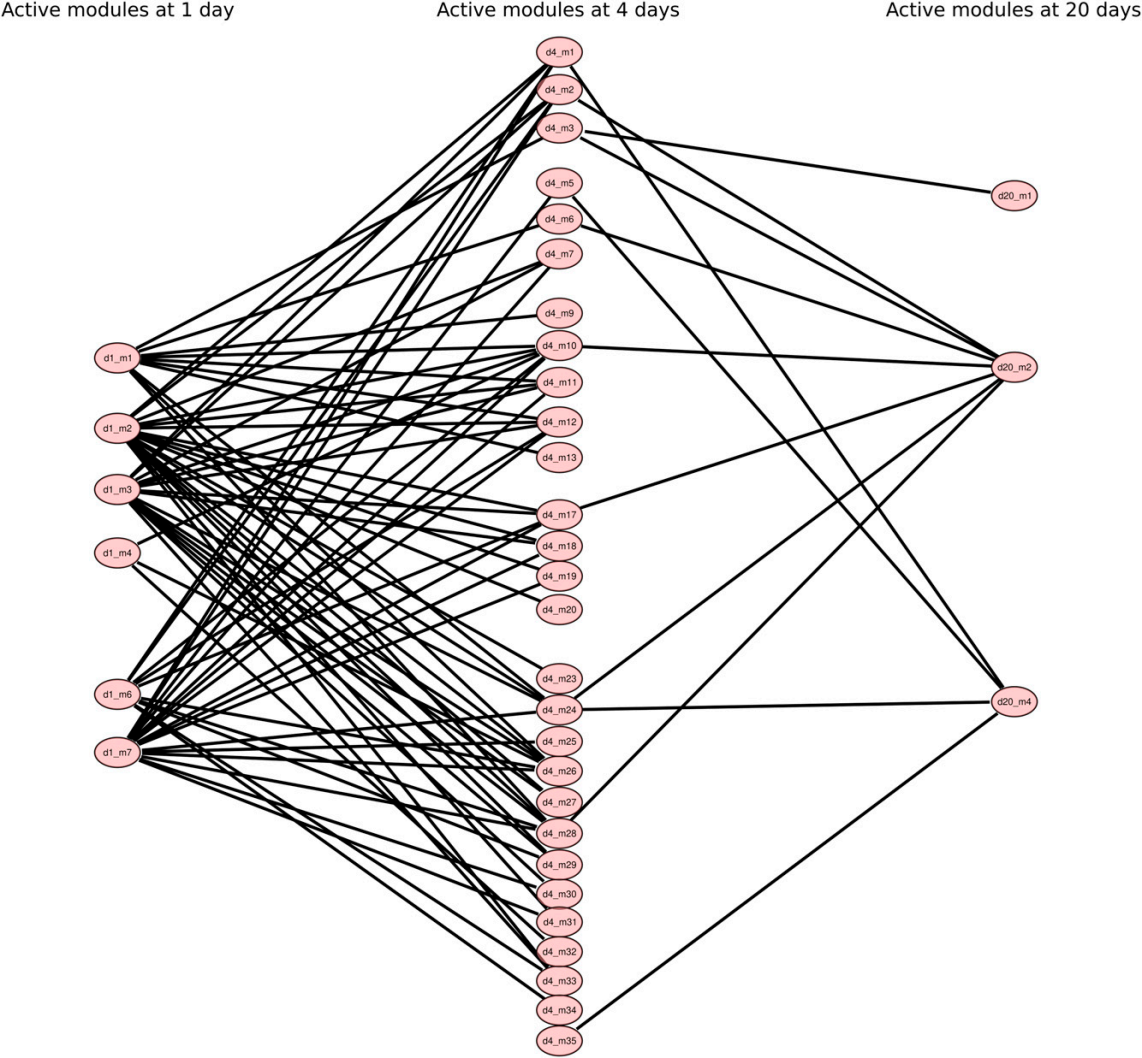
Interactions between modules at different time points. Visualization of the intermodule interactions in the form of a network in which each node is a module and each link represents an interaction between one or more genes of the corresponding modules. The modules are labeled with the time point followed by the number of modules. Thus, the module name “d4_m1” refers to module number 1 identified at day 4.

Similar to the findings from the differential analysis, the genes highlighted at each of the three time points were mostly different. This observation reinforces the hypothesis of successive waves of gene activation. To highlight interactions between modules at different time points, we used the same data for an exhaustive search: for each module we determined whether the interactors of the genes they contain are included in other modules (1-hop neighbors) or whether they interact with genes in other modules (2-hop neighbors). Fig 7 shows a visualization of the intermodule interactions in the form of a network in which each node is a module and each link represents an interaction between one or more genes of the corresponding modules. One can thus visualize a cascade of interactions in which most of the modules are involved.

### Enrichment analysis of active modules

The identification of active modules allows us to gain a clearer view of the studied process. On day 1, we retrieved a group of underexpressed genes enriched in functions related to cuticle development and chitin metabolic processes. The functions were already identified through a traditional enrichment analysis of differentially expressed genes, but in this study, we focused on a group of 22 genes in comparison to the 335 genes detected as underexpressed by DESeq2 (Love et al, 2014). We noted that despite its limited size, this module contains genes that cannot be detected via a standard differential analysis. FBgn0040582/BomBc3, FBgn0031940/CG7214, FBgn0032507/CG9377, and FBgn0033869/Cpr50Cb are indeed associated with FDR ranging from 0.10 to 0.90, which are well above the commonly used minimum threshold of 0.05.

Based on data collected by FlyAtlas, FBgn0040582/BomBc3 is very highly expressed in the adult head, heart, fat body, and carcass. This gene is also very highly expressed in female spermathecae of both virgin and mated females (Chintapalli et al, 2007). BomBc3 is known to control the Toll pathway, which plays a key role in the innate immune system. FBgn0031940/CG7214 has been implicated in the immune response (Pal et al, 2008). FBgn0032507/CG9377 is up-regulated in the brains of *Drosophila melanogaster* courting males compared with noncourting males (Ellis & Carney, 2011). All of these genes are highly expressed in the head fat body. Changes in expression in the fat body have been highlighted by a previous study in which the authors demonstrated that mating modifies fatty acid metabolism in the male brain (Ellis & Carney, 2010).

Among the other detected active modules, we found one module that contains overexpressed genes involved in the cell cycle and developmental processes, which are functions that have already been highlighted by the standard enrichment analysis procedure. This enrichment is also mostly related to the mitotic cell cycle and germ cell development and has been implied in metabolic processes. The third module was related to protein processing in the endoplasmic reticulum; the fourth module was involved in vitelline membrane formation; the fifth module was involved in the receptor signaling process; the sixth module was related to RNA polymerase II pretranscription events; and the seventh module was related to COPI-dependent Golgi-to-ER retrograde trafficking (Fig 3 and Table S9).

Table S9 List of enrichment of the active modules identified at days 1.

At day 4, we retrieved a large module (module 1 of Fig 4) containing overexpressed genes linked to rRNA processing and ribosome biogenesis. We found 37 other active modules enriched in genes with implied functions investigated in other studies, such as changes in the immune response (Kapelnikov et al, 2008a, 2008b – modules 6 and 13), lowering the expression of genes implied in the perception of smell (Lebreton et al, 2014 – module 14), midgut development (White et al, 2021 – module 29), and other processes that have been uncovered (Fig 4 and Table S10). At 20 d, the transcriptomes of virgin and mated females were quite similar because only four active modules were highlighted. Among these modules, we found genes involved in chitin metabolism that were also identified as being differentially expressed at day 1 (Fig 5 and Table S11). Surprisingly, the bioinformatically extracted modules were very specific for each considered period. Rare overlaps were observed between periods. However, when all the genes within the different modules at the three periods were assembled, significant interconnected modules were found. This finding suggests that a chronological and temporal order of activation/inhibition occurs after mating and continues during the female life span. At day 1, numerous early genes were affected, and the vast majority of these early genes returned to normal at day 4. These temporal and sequential gene networks that are manipulated by mating hint at internetwork regulation: the first wave orchestrates the second wave and so on down the cascade. Because the analysis was restricted to head tissue, the predicted genes involved in egg development, processing food uptake, and general metabolism were not considered “a priori.” However, the changes in the brain, which are likely related to changes in behavior, can be seen as considerable at days 1 and 4, although these changes appear to evaporate at the end of the life span.

Table S10 List of enrichment of the active modules identified at days 4.

Table S11 List of enrichment of the active modules identified at days 20.

### Comparison between AMINE, JActiveModules, ClustEx, DIAMOnD, and GiGA

The different programs were used to analyze the same data set, namely, the differential expression of genes at day 1. The execution of AMINE was performed as presented above. JActiveModules was executed using default parameters. ClustEx2 asks to specify the size of the largest module. There is a procedure to follow, described in the documentation, to determine the most appropriate size. Following the instructions, we have specified this size as 100. DIAMOnD generates a unique and ordered list of the most involved genes. The program asks for the size of this list which we set to 100. GiGA also uses the maximum module size as a parameter. The default value is 20; we have left it unchanged. On this dataset, AMINE finds seven modules ranging from 4 to 29 genes (Table S6), ClustEx2 finds 86 modules, of which some are very small and not really informative (Table S12). By keeping only the modules containing more than three genes, we obtain a list of 30 modules of size 4–100 (the maximum specified size). JActiveModules finds five modules (the number of modules specified in parameter) with size ranging from 23 to 228 (Table S13). DIAMOnD outputs an ordered list of 100 genes (Table S14). GiGA finds 21 modules of which two contain only two genes (Table S15). By keeping only the modules containing more than three genes, we obtain a list of 19 modules of size 4–19 (just below the specified maximum size).

Table S12 List of modules identified by ClustEx2 at day 1.

Table S13 List of modules identified by JActiveModules at day 1 and, for each module, the best GO enrichment with biological process terms.

Table S14 List of genes identified by DIAMOnD at day 1.

Table S15 List of modules identified by GiGA at day 1.

**Figure S1.**
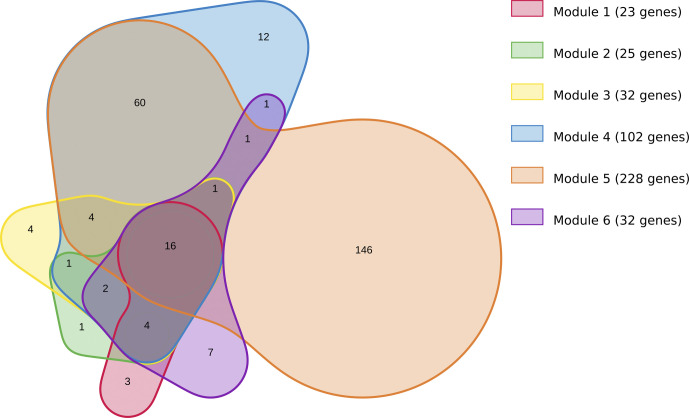
Euler diagram illustrating the overlap of the five modules identified by JActiveModules. On the diagram, each area represents one module found by JActiveModules. The sizes of the areas and the overlaps correspond to the sizes of the data sets. The figure was generated using the online tool nVenn, accessible at http://degradome.uniovi.es/cgi-bin/nVenn/nvenn.cgi (Pérez-Silva et al, 2018).

**Figure S2.**
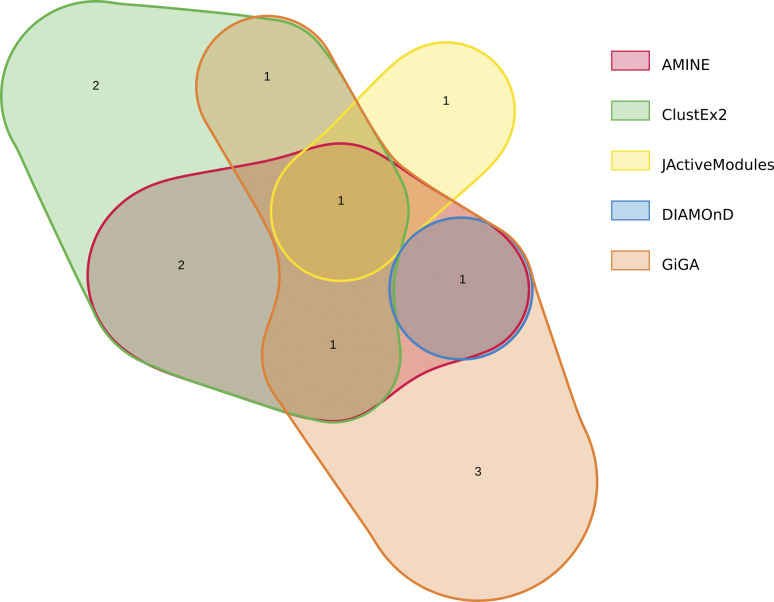
Euler diagram illustrating the overlap of the enrichment terms corresponding to the modules identified by AMINE, ClustEx2, JActiveModules, DIAMOnD, and GiGA. On the diagram, each area represents GO Biological Process enrichment for the modules revealed by the five methods. The sizes of the areas and the overlaps correspond to the sizes of the data sets. The figure was generated using the online tool nVenn, accessible at http://degradome.uniovi.es/cgi-bin/nVenn/nvenn.cgi (Pérez-Silva et al, 2018).

**Figure S3.**
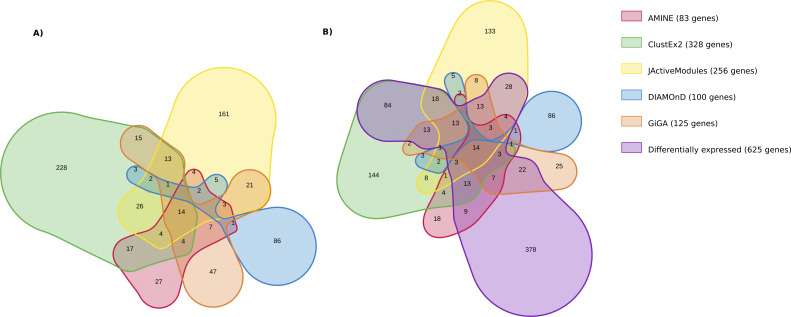
Euler diagram illustrating the overlap of the total of genes considered as important by several methods. On the diagram, each area represents the set of genes identified. The sizes of the circles and the overlaps correspond to the sizes of the data sets. The figure was generated using the online tool nVenn, accessible at http://degradome.uniovi.es/cgi-bin/nVenn/nvenn.cgi (Pérez-Silva et al, 2018). **(A)** Comparison between AMINE, ClustEx2, JActiveModules, DIAMOnD, and GiGA methods. **(B)** Comparison between the genes detected by the five module detection methods and those identified by differential expression analysis using a *P*-value less than 0.05 and a log_2_FC greater than 0.32 or less than −0.32.

The first thing we notice about jActiveModules is that although the method is able to identify several modules, they are very overlapping. Modules 1–3 contain a very similar group of genes. The content of modules 4 and 5 is more different, but mainly because these modules are larger. An Euler diagram displaying the five modules found by JActiveModules is shown in Fig S1. The GO enrichment with biological process terms corresponding to the genes within the identified modules are presented in Table S16. Not surprisingly, the first three modules identified by JActiveModules correspond to the same function which is “chitin-based cuticle development.” The two other modules correspond to “carbohydrate metabolic process.” The 100 genes highlighted by DIAMOnD correspond to the very generic function “cell cycle.” By selecting only the first genes appearing in the list, we obtain more specific functions: “cell cycle process” for the top 50 genes and “mitotic cell cycle process” for the top 25 genes. ClustEx2 and GiGA, like AMINE, generate non-overlapping gene modules. AMINE, ClustEx2, and GiGA identify a different number of modules but it is difficult to decide which is better for the user. We can gain an idea of the relevance of the modules by looking at which ones are significantly enriched in terms from GO biological process. Among the seven modules identified by AMINE, six are enriched with GO Biological Process with a FDR of less than 0.05 (which accounts for 85% of the total). It is also the case for 15 of the 30 modules identified by ClustEx2 (50% of the total) and 9 of the 19 modules identified by GiGA (47%). These ratios are not proof in themselves, but the fact that the identified modules correspond to known processes is rather a sign that coherent and functional modules have been identified. If we adopt a more global view by looking at the high-level GO terms, we obtain, for AMINE, modules corresponding to “cuticle development,” “cell cycle,” “transport,” “reproduction,” and “signaling.” For clustex2, 8 of the 15 modules found are associated with these same annotations except for “cell cycle,” for JActiveModules, three modules are associated with “cuticle development.” The set of genes highlighted by DIAMOnD are associated with “cell cycle” and GiGA reports modules associated with three annotations found by AMINE, 1 by ClustEx2, and 1 by DIAMOnD. In addition to the six main biological processes highlighted by AMINE, ClustEx2 identifies modules corresponding to “cell organisation,” “cellular metabolic process,” and “cell differentiation.” The two large modules identified by JActiveModules correspond to “carbohydrate metabolic process” and GiGA finds modules associated with: “cellular metabolic process,” “small molecule metabolic process,” “response to radiation,” and “system development.” Overall, all processes revealed by amine are shared with at least one other method. Two other annotations were identified only by ClustEx2, one only by JActiveModules and three only by GiGA (Fig S2). If we look at the set of genes considered, by each of the methods, as being important in the process studied, we notice that there is no gene in common between the five methods and that a significant proportion of genes is only found by only one method (Fig S3A). However, this proportion of genes only identified by one method differs; it is 32% for amine, 36% for GiGA, 62% for JActiveModules, 69% for ClustEx2, and 86% for DIAMOnD. Overall, we note that the AMINE method identifies the fewest genes but has the most genes found in common with each of the other methods. If we compare with the genes identified by differential expression analysis using a *P*-value less than 0.05 and a log_2_FC greater than 0.32 or less than −0.32, we find that most of the genes with significant variation are not highlighted by either of the module detection methods. Conversely, 443 genes are only identified by module detection methods (Fig S3B). This means that all the reviewed module detection methods have the ability to pinpoint genes that may have an influence on the studied process but whose expression variations are very small (a log_2_FC of 0.32 corresponds to a variation of ±25%).

Table S16 GO enrichment with biological process terms corresponding to the genes within the modules identified at day 1 by AMINE, ClustEx2, DIAMOnD, and JActiveModules.

**Figure S4.**
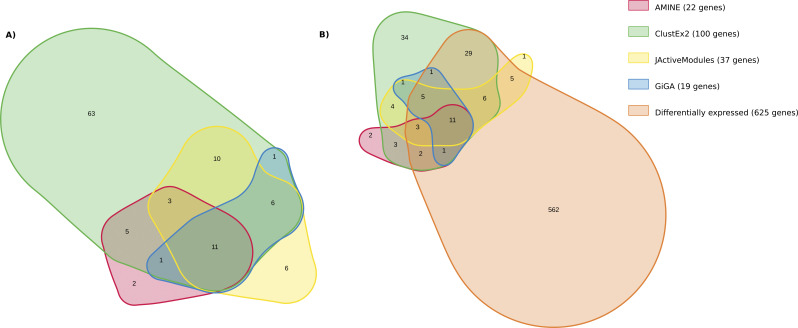
Euler diagram illustrating the overlap between the genes belonging to modules associated with “chitin-based cuticle development” by AMINE, ClustEx2, JActiveModules, and GiGA. On the diagram, each area represents the set of genes identified. The sizes of the circles and the overlaps correspond to the sizes of the data sets. The figure was generated using the online tool nVenn, accessible at http://degradome.uniovi.es/cgi-bin/nVenn/nvenn.cgi (Pérez-Silva et al, 2018). **(A)** Comparison between AMINE, ClustEx2, JActiveModules, and GiGA methods. **(B)** Comparison between the genes detected by the four module detection methods and those identified by differential expression analysis using a *P*-value less than 0.05 and a log_2_FC greater than 0.32 or less than −0.32.

The Biological Process “chitin-based cuticle development” is an important process occurring at day 1. This annotation has been highlighted both by differential gene analysis and by four out of five module detection methods. AMINE found a cluster of 22 genes implied in this process, ClustEx2 highlighted a cluster of 100 genes, JActiveModules detected three overlapping modules representing a total of 37 genes, and GiGA found a cluster of 19 genes. Fig S4A shows the overlap between the modules associated with “chitin-based cuticle development” for the four different methods. Differential expression analysis reveals 625 genes with a log_2_FC greater than 0.32 or less than −0.32. Of these 625 genes, 63 (representing 10%) were also identified by one or more of the module detection methods. However, even when using a log_2_FC threshold as low as 0.32, 45 genes were only revealed using module detection methods. This again emphasizes that searching for groups of genes working together can pick out important genes that move very little during the experiment. The overlap between differentially expressed genes and modules associated with “chitin-based cuticle development” for AMINE, ClustEx2, JActiveModules, and GiGA is shown in Fig S4B. This illustrates that only using methods that combine both the differential expression of genes and their interactions can relate the process under study to particular genes that vary only slightly.

This comparative analysis on a real-world dataset highlights the advantages of AMINE over the few other easy-to-use methods. First of all, it does not require installation because it can be run via a web interface. It does not require any special formatting of the input data because it is sufficient to provide tabulated or Excel files generated by differential analysis pipelines. The use of AMINE is particularly simple because no parameters are required, unlike the other four tested methods which requires either the number of modules to search or the maximum size of the modules. In terms of results, they are rather more concise than the other methods because the modules are less numerous or contain fewer genes. This conciseness allows biologists to focus on a smaller number of genes that appear to be highly relevant to the process being studied because a large proportion of them are also identified by other methods.

## Discussion

Changes in gene expression in the female head appear unique at different time scales after mating that fills spermathecae with sperm and other molecules in the seminal fluid. These changes appear to be a continued developmental program occurring in adulthood. The *Drosophila* female phenotypic plasticity after mating is remarkable, as stimuli released and/or contained in sperm might orchestrate a programmatic outcome that underlies the transcriptomic temporal changes in mated female flies. Changes in the behavior of mated versus virgin female flies could be seen as an extension of phenotypic plasticity, and from this point of view, caste distribution in eusocial insects could be considered as the most spectacular and achieved modality. Sperm acquisition, as any environmental change that faces the *Drosophila* female, can alter some physiological functions and behavior over short and long time scales. Our analysis unraveled the transcriptomic modifications in the *Drosophila* model through which the genome shapes diverse physiological functions in relation to metabolism needed for fertilized egg laying. Unrelated to the genetic coding of spermatozoids, the effect of male seminal fluid proteins on the post-mating physiology and behavior of females has been documented in the past. The implicated molecules likely include peptides, proteins, small molecules, and RNAs that act to promote profound behavioral changes regarding oviposition choices, changes in feeding behavior and mating refractoriness in females (McGraw et al, 2004, 2008).

Mating-induced transcriptome changes in females have been also reported in diverse tissues or the entire body of many insects, including *D. melanogaster* (Lawniczak & Begun, 2004; McGraw et al, 2004, 2008; Innocenti & Morrow, 2009; Short & Lazzaro, 2013), *Apis mellifera* (Kocher et al, 2008), *Ceratitis capitate* (Gomulski et al, 2012), and *Anopheles gambiae* (Rogers et al, 2008). To this regard, the transcriptomic changes in the oviduct of *D. melanogaster* and *Aedes aegypti* have been extensively studied (Kapelnikov et al, 2008a, 2008b; Alfonso-Parra et al, 2016). The kinetics of the published RNA-Seq lists show that in these later species, few genes are affected after a few hours of copulation, whereas maximal gene expression changes are observed at 6 h (Heifetz et al, 2001, 2014; Heifetz & Wolfner, 2004; Carmel et al, 2016). In honeybee, the mating process induce marked changes in the expression of genes related to vision, chemoreception, metabolism, and immunity (Fahrbach et al, 1995; Richard et al, 2007; Kocher et al, 2009). In contrast, reverting the transcriptome from mated females back to virgin females has not been observed in honeybee species in which distinct phenotypes emerge from the same genotype, giving caste attributes to individuals (Page & Peng, 2001). The gene expression levels in the full-scale genome of eusocial insects like honeybees and aunts has provided the evidence for the irreversible programmatic changes associated with mating in mated versus the virgin queen. (Whitfield et al, 2003; Grozinger et al, 2007; Simola et al, 2016). The transcriptomic changes map known behavioral and physiological traits that characterize the transition from a virgin queen to a newly mated queen. If most queens start laying eggs after one mating flight, others will engage in multiple mating flights with a new set of multiple males at each time, which enrich the seminal male compounds in relation to their genetic background (Woyke, 1964; Tarpy & Page, 2000). More interestingly, virgin queens are photophilic (Kocher et al, 2010) and aggressive towards other virgin queens (Manfredini et al, 2015). In contrast, mated queens are photophobic, lack aggressiveness, and move protected by clustered workers (Kocher et al, 2010; Manfredini et al, 2015). In social Hymenoptera (ants, some bees, and wasps), mated queens that present extended lifespans compared with nonreproductive workers or virgin queen show the same physiological and behavioral changes, which triggers a nest-bound egg-laying status (Page & Peng, 2001; Heinze & Schrempf, 2008; Castella et al, 2009; Simola et al, 2016). However, for most of insect species for which this phenomenon has been reported, the molecular mechanisms remain unelucidated because of the lack of powerful genetic tools for silencing genes.

In contrast, significant advances have been obtained with the *Drosophila* model for which the seminal fluid protein sex peptide (SP) was found to activate an identified receptor in female fly, which constitutes one rare known pathway in insect world (Yapici et al, 2008). Defects in this seminal fluid signaling process are hypothesized to provoke reproductive disorders in females, which eventually results in reduced offspring output or unhealthy offspring or, in an ultimate scenario, a reproductive barrier that might guide or lead to speciation (Delbare et al, 2017). Among the other known molecules in *Drosophila*, the seminal fluid proteins (Acps) produced by male accessory glands were found partly responsible for the post-mating transcriptomic changes (Kubli, 2003; Swanson, 2003; McGraw et al, 2004; Peng et al, 2005; Isaac et al, 2010). More than 100 potential Acps have been identified in male *Drosophila sperm* as potential candidates to induce changes in tissue specific transcriptome and a lack of one of them, SP (Acp70A), in seminal fluid is correlated with a reduced number of laid eggs (Kubli, 2003; Swanson, 2003), which triggers female remating (Chapman et al, 1996, 2003; Liu & Kubli, 2003) and lowers female food uptake (Carvalho et al, 2006). The same way, Acp29AB and Acp62F were found responsible for a large panel of modified genes in mated female (McGraw et al, 2004).

Although modified gene expression is expected to refer to egg formation and is observed in ovaries and reproductive tracts, researchers have noted that marked transcriptional changes occur in the brain, fat body, or other tissues (McGraw et al, 2008; Mack, et al, 2006; Kapelnikov et al, 2008a, 2008b; Prokupek et al, 2009). Data obtained using microarray techniques have revealed a transcriptional response to mating with a peak of intensity after 3 h and a sharp decline at later time points (McGraw et al, 2008). Other researchers using the same technology have confirmed a peak at 6 h with changes that involve more than 500 genes (Mack, et al, 2006; Kapelnikov et al, 2008a, 2008b; Prokupek et al, 2009). Genetic manipulation that causes defects in ejaculation have allowed to identify molecules involved in post-mating modifications and the role of individual seminal factors for each specific female physiological trait (Chapman et al, 2003, 1996; Liu & Kubli, 2003; Wigby & Chapman, 2005; Wigby et al, 2011).

A comprehensive review outlines the seminal proteins that have been identified in many insect species for which the physiological functions in relation with female post-mating responses remain elusive (Avila et al, 2011). However, to disentangle the roles of spermatozoid component, the seminal Acps and other active molecules in seminal fluid will be a very challenging task for most of insect species.

In *Drosophila*, integrative signaling molecules present in seminal fluid coordinate global homeostasis and physiology involving the female nervous system, fat body, endocrine cells, gut/microbiome, and reproductive tissues. The role of seminal fluid as a contributor to the fine regulation in a continuum with the action of the couple juvenile hormone/ecdysone in young adult females has been investigated in the past on the basis of genetic screen of mutants (Toivonen & Partridge, 2009; Rajan & Perrimon, 2011; Droujinine & Perrimon, 2013, 2016).

In this report, we have restrained our analysis to female brain transcriptional modification induced by mating. We found that only two post-mating time periods presented considerable scale changes at 1 d (625 genes), which declined at 4 d (82 genes) to end at 20 d, when only a few genes showed lasting expression changes. Amazingly, most of the identified genes showing modified expression at each stage were unique with little, if any, overlap with those identified at other stages. This finding argues in favor of time-specific activation/inhibition of gene expression organized by the recruitment of successive waves of interconnected genes that occurs after mating. Obviously, the first cascade of changes induces specific changes in the second cascade, and so on up to the end of the process. The most striking fact is that each wave presents a unique set of implicated genes. Unfortunately, it is difficult to characterize these interaction cascades more precisely given the data used in the study. Indeed, we only have data obtained at 1, 4, and 20 d, but many changes, including the activation of other gene modules that cannot be detected, can occur between these time points.

It is clear that signaling molecules in seminal liquid and/or within spermatozoid cells act at distance through unknown processes, such as the hemolymph transport of soluble compounds or microvesicles traveling with RNA and lipids, to regulate egg production, homeostasis, and behavior. Many reports in the literature have described the molecular transport between tissues as intertissue dialogue, which attributes credibility to the present scenarios (Rajan & Perrimon, 2011; Droujinine & Perrimon, 2013, 2016). In this regard, this field of investigation has achieved solid advances in mammals where extracellular vesicles containing RNA molecules have been shown to be transported through the blood–brain barrier, which results in the delivery of functional RNAs to brain neurons, the origin of these RNAs being far remote, mainly located in peripheral tissues such as the genital tractus (Rassoulzadegan et al, 2006; Gapp et al, 2014; Skinner et al, 2015; Chen et al, 2016a, 2016b).

Recent findings have proven that RNAs are capable of conferring information regarding interphase environment/germline interactions to guide, to some extent, the destiny of offspring through modification of developmental processes (Rassoulzadegan et al, 2006; Gapp et al, 2014; Skinner et al, 2015; Chen et al, 2016a, 2016b). Recently, robust experimental data have documented a direct causal role of sperm RNAs in transferring deleterious phenotypic traits across generations in mammals, which adds a new paradigm to an already well documented heredity driven by chromosomal DNA mutation insertions/deletions that generate allele catalogs (Rassoulzadegan et al, 2006; Gapp et al, 2014; Skinner et al, 2015; Chen et al, 2016a, 2016b). These studies in mammals might suggest that similar scenario might occur in insects like *Drosophila* where seminal compounds including RNAs could manipulate the phenotypes of offspring in parallel with their action on mated female brain. If “memorized” sperm RNA or other molecules turn to be epigenetic markers, we might hypothesize that the female *Drosophila* responses to sperm signals could be co-substantial to phenotypic changes in some offspring traits in a non-Mendelian manner as a response to life history and environmental cues experienced by parents.

Understanding how mating during *Drosophila* reproduction triggers a developmental program extension in the female life span constitutes conceptual and experimental challenges to understand the insect reproduction biology.

## Materials and Methods

### Mating protocol and fly maintenance

Flies were raised at 25° under 16:8 h light–dark cycle and kept in bottles with standard cornmeal food media. For the mating protocol, the emerged flies were collected on short period of time (1 h) immediately after eclosion. Males and females at 1:1 ratio were left for 24 h together in fresh bottles of food to achieve 100% mating. Females were then removed and placed in fresh bottle for 1, 4, and 20 d. Fly heads were isolated and anesthetized by C02 and flash frozen in liquid nitrogen for 1 min. Male and female were separated. The frozen heads were cut off with a dissecting forceps. A group of 100 flies were processed for each determination. The biological material were stored at −80°C until the RNA was purified. See Brown et al (2014) and Graveley et al (2011) for more details.

### Differential expression analysis

Data of gene expression in the head of both virgin and mated *Drosophila* females made available by the modENCODE project were downloaded as FASTQ files from the Sequence Read Archive with the accession ID: PRJNA75285. For the RNA isolation, Illumina RNA-Seq library construction and sequencing, Read mapping and filtering, Differential Gene Expression Analysis, and Statistical methods are developed in supplemental information of the two related canonical articles (Graveley et al, 2011; Brown et al, 2014). The data contain the results from RNA-Seq experiments performed at three different time points: 1, 4, and 20 d. Details of these datasets are presented in Table S17.

Table S17 Details of the data analyzed.

The transcript abundance was quantified with Salmon (Patro et al, 2017), and the differential gene expression at each time point was calculated using DESeq2. Genes were considered differentially expressed if they showed a variation of at least a factor of 1.25 (i.e., a log_2_-fold change greater than 0.32 or less than −0.32) associated with an adjusted *P*-value of at most 0.05.

### Enrichment analysis of differentially expressed genes

Functional enrichments for Gene Ontology terms, KEGG and Reactome Pathways, protein domains and UniProt Keywords were retrieved using Cytoscape (Shannon et al, 2003) and the StringApp plugin (Doncheva et al, 2018). The annotations identified with an FDR of less than 0.05 are listed in Table S4.

### Identification of active modules

**Figure 8.**
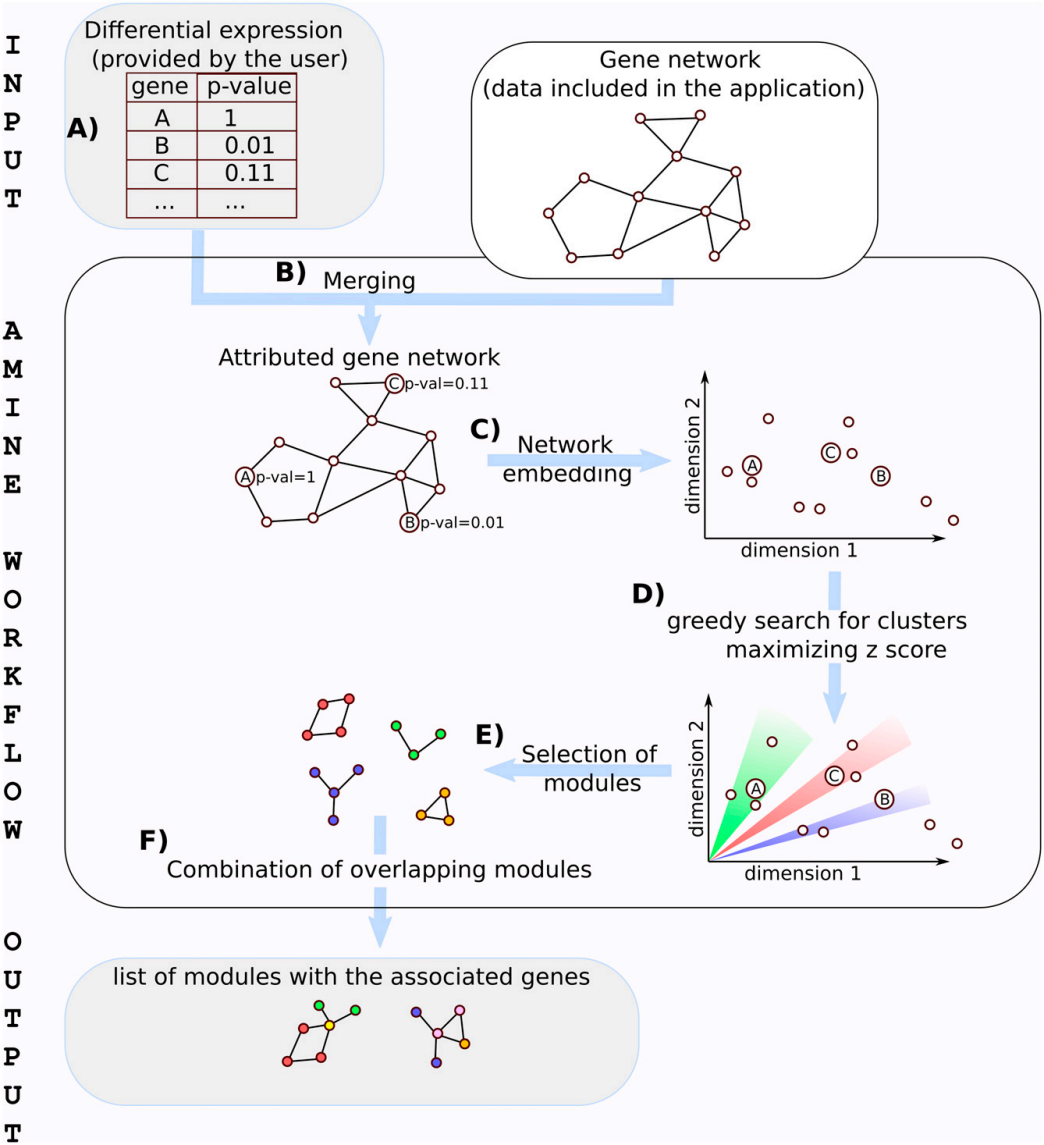
Workflow of the AMINE method. **(A)** Input data are composed of a table storing the significance of the expression variation of genes between two conditions and a network representing known gene interactions. Gene variations must be provided by the user, whereas data concerning gene interactions are included in the application. **(B)** Data about gene interactions and gene variations are merged to generate an attributed gene network. **(C)** Nodes belonging to the attributed gene network are mapped to a low-dimensional space through the use of a biased Node2Vec method. **(D)** Sets of genes that are both cohesive and differentially expressed are identified in the embedded space by maximizing both the scores of the nodes and the cosine distance between the vectors representing the nodes. **(E)** Each gene from the gene network is associated with the cluster that maximizes the z-score. **(F)** Redundancy in the content of modules is ruled out by combining sets of nodes obtained in the previous step while ensuring that the result remains spatially cohesive.

The search for active modules has been performed with AMINE (Pasquier et al, 2021 *Preprint*). The AMINE method was designed to identify the modules of genes that are triggered in a biological experiment. It uses as input the background knowledge on the interactions between genes and measurements representing, in the specific context of a given experiment, indicators of the involvement of genes in the studied process. Gene interactions data are retrieved from the String database (Szklarczyk et al, 2017) with a combined evidence score ≥0.7 and included in the application. Measurements on the activity of genes must be supplied by the user. They are represented by the *P*-value, adjusted *P*-values, or FDR associated with the fold changes of the genes. At a minimum, the data are composed of a tabular file consisting of two columns: one containing the name of the gene and the other, the associated *P*-value. A more convenient way to specify the data to be used is to simply input the result file produced by differential expression analysis methods such as DeSeq2 or EdgeR (Robinson et al, 2010). The input file can be in csv (comma-separated values) or xlsx format (Fig 8A).

The interaction data are merged with gene associated *P*-values to generate an attributed gene network in which vertices represent genes, edges represent interactions between genes, and each vertex is annotated with a numeric attribute reflecting its associated *P*-value (Fig 8B).

The method relies on Node2vec (Grover & Leskovec, 2016) to generate a highly informative and compact vector representation for each vertex in the network. This transformation is called network embedding (Fig 8C). In the resulting vector space, the cosine distance between the vectors representing the nodes accurately reflect their proximity in the original network. AMINE, then, uses a greedy approach to build, from each vertex, groups of vertices of increasing size based on the aggregation of the closest vertices; the closeness being calculated with the cosine distance of the vertices' encoding vectors. Each group of vertices is then evaluated using Stouffer’s Z method (Ideker et al, 2002). To do this, we first transform the *P*-value associated with each vertex into a z-score. The z-score *z*(*v*_*i*_) corresponding to the vertex *v*_*i*_ associated with the *P*-value *p*_*i*_, is calculated with *z*(*v*_*i*_) = *Φ*^−1^(1 − *p*_*i*_), where *Φ* is the standard normal cumulative distribution function. Let *lov*(*v*_*i*_) be the list of vertices in the network sorted by their cosine distance from *v*_*i*_ (closest vertices fist, which implies that *v*_0_ = *v*_*i*_), the aggregate z-score *z*(*v*_*i*_, *k*) for a set of nodes composed of the *k* closest vertices of *v*_*i*_ in the vector space is computed with:

$$z\left(v_{i},k\right)=\frac{1}{\sqrt{k+1}}\sum_{j=0}^{j<k+1}z\left(\mathrm{lov}\left(v_{i}\right)\left[j\right]\right)$$

We get the best cluster associated to each vertex by selecting *k* such that it allows us to obtain the best z-score (Fig 8D and E).

The last phase of the method consists in combining the different clusters while ensuring that new merged clusters remain spatially cohesive (Fig 8F). We say that two clusters *M*_*i*_ and *M*_*j*_ are spatially cohesive when *M*_*i*_ ∩ *M*_*j*_ ≠ ∅. Starting from the module with the higher z-score, the process consists in evaluating all possible clusters formed by *M*_*i*_ ∪ *℘*(*M*_*j*/*j*≠*i*_) using the z-score, with *℘*(*M*_*i*_) denoting the powerset of *M*_*i*_ and keeping the modules with the highest z-score. The workflow of the AMINE method is presented in Fig 8.

The details of the method and the demonstration of its effectiveness are described in an article by Pasquier et al (2021) *Preprint*. The great advantage of AMINE is that it does not require any settings; it is not even necessary to indicate the number of modules to be identified or the size of the modules.

### Data access

The transcriptomic datasets were retrieved from the Sequence Read Archive: https://www.ncbi.nlm.nih.gov/sra with accession numbers SRR070436, SRR070437, SRR100281, SRR070430, SRR100278, SRR100282, SRR070388, SRR070419, SRR100275, SRR070434, SRR070435, SRR100279, SRR070414, SRR070415, SRR111882, SRR070420, SRR116383, and SRR100274.

## Supplementary Material

Reviewer comments
