# Supplementary material for: Temporal and sequential order of nonoverlapping gene networks unraveled in mated female Drosophila
Source: Life Sci Alliance. 2021 Nov 29;5(2):e202101119. doi: 10.26508/lsa.202101119 (PMC8645335; doi:10.26508/lsa.202101119)
Supplement: Supplementary file 17 [file LSA-2021-01119_TableS17.docx]

Table S17. Details of the data analyzed. The table shows the accession number, a description, the number of spots and the number of bases for each of the 18 runs.

| Run | Description | # of spots | # of bases |
| --- | --- | --- | --- |
| SRR070436 | Adult Virgin Female, 1 day Post-eclosion, Head | 2.9G | 18,315,927 |
| SRR070437 | Adult Virgin Female, 1 day Post-eclosion, Head | 3.0G | 18,799,049 |
| SRR100281 | Adult Virgin Female, 1 day Post-eclosion, Head | 21 G | 105,072,963 |
| SRR070430 | Adult Virgin Female, 4 days Post-eclosion, Head | 4G | 24,772,788 |
| SRR100278 | Adult Virgin Female, 4 days Post-eclosion, Head | 3.1G | 20,418,604 |
| SRR100282 | Adult Virgin Female, 4 days Post-eclosion, Head | 17G | 84,868,661 |
| SRR070388 | Adult Virgin Female, 20 days Post-eclosion, Head | 2.4G | 15,567,474 |
| SRR070419 | Adult Virgin Female, 20 days Post-eclosion, Head | 3.3G | 20,998,027 |
| SRR100275 | Adult Virgin Female, 20 days Post-eclosion, Head | 3.5G | 23,341,590 |
| SRR070434 | Adult Mated Female, 1 day Post-eclosion, Head | 4G | 25,284,594 |
| SRR070435 | Adult Mated Female, 1 day Post-eclosion, Head | 3.9G | 24,475,616 |
| SRR100279 | Adult Mated Female, 1 day Post-eclosion, Head | 17.4G | 86,766,586 |
| SRR070414 | Adult Mated Female, 4 days Post-eclosion, Head | 5.9G | 38,050,589 |
| SRR070415 | Adult Mated Female, 4 days Post-eclosion, Head | 6.9G | 44,272,369 |
| SRR111882 | Adult Mated Female, 20 days Post-eclosion, Head | 2.1G | 13,953,381 |
| SRR070420 | Adult Mated Female, 20 days Post-eclosion, Head | 1.4G | 9,051,901 |
| SRR116383 | Adult Mated Female, 20 days Post-eclosion, Head | 1.2G | 8,103,005 |
| SRR100274 | Adult Mated Female, 20 days Post-eclosion, Head | 1G | 6,805,540 |
